# Can Platelet Count and Mean Platelet Volume and Red Cell Distribution Width Be Used as a Prognostic Factor for Mortality in Intensive Care Unit?

**DOI:** 10.7759/cureus.11630

**Published:** 2020-11-22

**Authors:** Mehmet Duran, Öznur Uludağ

**Affiliations:** 1 Anesthesiology and Reanimation, Adiyaman University Education and Research Hospital, Adiyaman, TUR; 2 Anesthesiology and Reanimation, Adıyaman University Faculty of Medicine, Adıyaman, TUR

**Keywords:** mortality, mean platelet volüme, platelet volüme, red cell distribution width, comorbid disease

## Abstract

Introduction

Critical patients are those patients who have psychological unstability that can cause morbidity and mortality in a short period of time. These patients need to be intensively monitored for organ function like cardiovascular, respiratory and neurological system. The most critical patients are transferred to intensive care unit (ICU) for close watch. It is not rare that hematological system of critical patient is affected from strong inflammation.

The main purpose of this study is to be able to determine platelet count (PLT), mean platelet volume (MPV) and red cell distribution width (RDW) admission value to predict mortality in ICU. Secondary purpose is to present a view about clinical use of these blood parameters.

Material and methods

In this study, RDW, MPV and PLT values of the patients in the first intensive care admission were evaluated and were compared with the last hemogram values before death. Glasgow Coma Score (GCS) and other risk factors for mortality were tried to be determined to show determinants of scoring systems on mortality in patients admitted to ICU.

Results

When compared with ICU entry in all patient groups and laboratory markers prior to exitus, the value of the input RDW was 14.66 ± 3.08 and the output RDW was 15.94 ± 9.59. Admission value of MPV was 8.180 ± 2.09, and before death the value of MPV was 9.199 ± 2.24. Statistically, it was significantly high (p < 0.001). The MPV values increased in all groups and cerebrovascular disease (CVD), respiratory failure, cardiac causes, head trauma and malignancies were statistically significantly high (p < 0.05). Admission value of PLT was 215.46 ± 116.8, and before death the value of PLT was 154.73 ± 101.32. Statistically, it was significantly low (p < 0.001).

Conclusions

The study showed that the difference between PLT, MPV and RDW values in the ICU and values before death, and decrease in PLT and increase in MPV and RDW in all patients were statistically significant. We believe that decrease in PLT, increase in MPV and RDW is a prognostic factor for mortality.

## Introduction

Intensive care unit (ICU) is the place where patients with life-threating diseases are kept for close watch. The demand of intensive care patients’ bed is gradually increasing and it is associated with increased length of life and population. Various scoring systems are used to predict mortality and prognosis of ICU patients and to evaluate and improve clinical research and treatment [1]. Strong inflammation affects hematological system of critical patient widely.

Hematological profiles like hematocrit, white blood cell and platelet are used in many accepted organ failure assessment score such as sequential organ failure assessment (SOFA) score, multiple organ dysfunction score (MODS), logistic organ dysfunction score (LODS) [2-4]. Platelet count (PLT) changes with inflammation. Mean platelet volume (MPV) can reflect platelet function better than platelet count test. Bigger platelets have more granule and prothrombotic particles [5]. Red cell distribution width (RDW), which shows erythrocyte morphology, is usually used in differential diagnosis of anemia. In addition, systemic inflammation, malnutrition, ineffective erythropoiesis and bone marrow dysfunctions can cause to increase RDW too [6]. These laboratory tests have become very popular because of low cost and easy accessibility in critical practice.

In studies up to now, generally the relationship between diseases and only one value of RDW, PLT and MPV was analyzed. As far as we know, the relationship between the values of ICU admission and the values before death was not investigated for the first time in mortality and different patient groups. Therefore, we hypothesized that RDW, PLT and MPV changes could reflect acute disease states and could provide more prognostic information than basal RDW, PLT and MPV alone.

## Materials and methods

In this study, after the approval of the Ethics Committee of Adiyaman University Medical Faculty, the files of 402 patients, who died from the group of 2116 patients hospitalized in Adiyaman University Education and Research Hospital between January 2014 and December 2015, were investigated retrospectively.

The demographic characteristics of the patients like age and gender, Glasgow Coma Score (GCS), primary diagnosis requiring ICU hospitalization, number of comorbidities, duration of intensive care unit stay and duration of mechanical ventilation were viewed.

GCS (eye, verbal response and motor response were evaluated, values between 3-15) was calculated according to the neurological examination findings at the time of admission to the intensive care unit. Twelve patients were excluded from the study because there were no blood samples before death. Five patients with chronic renal failure were excluded from the study.

Patients who died within 48 hours of their admission, patients younger than 18 years, patients with burn, patients with hematological malignancy (acute lymphoblastic lymphoma, acute myelodysplastic lymphoma, etc.), patients going to receive chemotherapy soon, patients undergoing platelet transfusion, acute myocardial infarction and cardiovascular surgery were excluded from the study. A total of 293 patients were included in the study.

RDW, MPV and PLT values ​​of the patients in the first intensive care admission were evaluated and were compared with the last hemoglobin values ​​before death. GCS and other risk factors for mortality were tried to be determined to show determinants of scoring systems on mortality in patients admitted to ICU.

Biochemical tests

Drawing blood sample for MPV measurement was performed before and during treatment in all cases. Blood samples were taken into standard tube containing ethylenediaminetetraacetic acid (EDTA) and stored at room temperature. All measurements were performed within 30 minutes of drawing blood using Beckman Coulter LH 780 Haematology System (Beckman Coulter Inc., Brea, CA). According to our laboratory, normal PLT values ​​were 154.00-400.00 K/uL, normal MPV values were ​​6.9-10.8 fl and RDW values ​​were accepted as 11.7-14.4%.

Statistical analysis

The frequencies, rates, mean and standard deviations of the patients in terms of different variables were presented as descriptive statistics. T-tests for independent groups in paired comparison groups, were run and mean ± standard deviation values were reported. Categorical variable Chi-square analysis was conducted to examine the differences between the ratios of distributions to groups, and number and percentage values were reported. The significance level for the analysis results was P < 0.05 determined. For analysis of data in this study, SPPS version 16 (SPSS Inc., Chicago, IL) was used.

## Results

The data of 402 (19%) patients, who died from the group of 2116 patients admitted to ICU in a two-year period, were analyzed retrospectively and 293 patients were included in the study. A total of 170 (58%) patients were male and 123 (42%) patients were female. The mean age was 67.29 ± 19.4. Mean duration of hospitalization was 13.56 ± 16.8 day. Mean duration of mechanical ventilator was 9.98 ± 12.2 day. The mean GCS recorded at admission was 7.06 ± 2.21. When compared with ICU entry in all patient groups and laboratory markers prior to exitus, the value of the input RDW was 14.66 ± 3.08 and the output RDW was 15.94 ± 9.59. Statistically, it was significantly high (p = 0.001). Admission value of PLT was 215.46 ± 116.8, and before death the value of PLT was 154.73 ± 101.32. Statistically, it was significantly low (p < 0.001). Admission value of MPV was 8.180 ± 2.09, before death the value of MPV was 9.199 ± 2.24. Statistically, it was significantly high (p < 0.001) (Table 1).

**Table 1:** Comparison of laboratory markers before ICU entry and exitus *p < 0.05, RDW: Red cell distribution width, PLT: Platelet, MPV: Mean platelet volume

	Entry values	Before exitus values	p-value
RDW (%)	14.66 ± 3.08	15.94 ± 9.59	0.001*
PLT (/mm^3^)	215.46 ± 116.8	154.73 ± 101.32	0.000*
MPV (fL)	8.180 ± 2.09	9.199 ± 2.24	0.000*

RDW values increased in all groups according to hospitalization and were significantly higher in respiratory failure, cardiac causes, other than head trauma, malignancy and sepsis (p < 0.05) (Table 2).

**Table 2:** RDW value by diagnosis CVD: Cerebrovascular disease, RDW: Red cell distribution width

	n	Entry RDW	Before exitus RDW value	p-value
CVD	101	13.91 ± 2.7	16.35 ± 15.9	0.156
Respiratory failure	69	15.59 ± 2.98	15.96 ± 2.71	0.000*
Cardiac causes	27	15.42 ± 3.25	16.27 ± 3.1	0.000*
Head trauma	28	12.89 ± 2.42	14.79 ± 2.82	0.467
Other trauma	13	14.09 ± 5.67	15.57 ± 3.62	0.014*
Malignancy	32	15.4 ± 2.82	15.87 ± 2.39	0.000*
Sepsis	23	15.65 ± 2.18	15.41 ± 2.26	0.000*

MPV values ​​increased in all groups and cerebrovascular disease (CVD), respiratory failure, cardiac causes, head trauma and malignancies were statistically significantly high (p < 0.05) (Table 3).

**Table 3:** MPV value by diagnosis CVD: Cerebrovascular disease, MPV: Mean platelet volume

	n	Entry MPV	Before exitus MPV value	p-value
CVD	101	8.35 ± 1.88	9.5 ± 2.15	0.000*
Respiratory failure	69	8.6 ± 1.92	9.4 ± 2.2	0.000*
Cardiac causes	27	8.22 ± 3.1	9.28 ± 2.74	0.000*
Head trauma	28	7.28 ± 1.23	8.55 ± 1.85	0.000*
Other trauma	13	7.07 ± 1.9	8.05 ± 1.6	0.073
Malignancy	32	7.98 ± 2.25	8.98 ± 2.55	0.000*
Sepsis	23	8.09 ± 2.78	8.78 ± 2.18	0.298

PLT values ​​decreased in all groups and were significantly high in CVD, respiratory failure and malignancies (p < 0.05) (Table 4).

**Table 4:** Platelet value by diagnosis CVD: Cerebrovascular disease, PLT: Platelet

	n	Entry PLT	Before exitus PLT value	p-value
CVD	101	232.38 ± 105	170.3 ± 102.2	0.001*
Respiratory failure	69	199 ± 93.9	152.4 ± 97.3	0.000*
Cardiac causes	27	202.5 ± 103.7	159.6 ± 118.5	0.653
Head trauma	28	222.9 ± 117.4	143.8 ± 117.4	0.936
Other trauma	13	257.3 ± 227.2	175.1 ± 94.2	0.065
Malignancy	32	194 ± 125.2	119.1 ± 93.9	0.002*
Sepsis	23	202.7 ± 138.7	138.7 ± 96.6	0.655

## Discussion

There are two important findings in our study. Firstly, decrease in PLT, increase in MPV and RDW values ​​were statistically significant in all groups. Secondly, the PLT values decreased in all groups ​​according to the hospitalization, and were statistically significantly low in CVD, respiratory failure and malignancies. MPV values ​​increased in all groups, and were statistically significantly high in CVD, respiratory failure, cardiac causes, head trauma and malignancies.

RDW values increased in all groups and were significantly high in respiratory failure, cardiac causes, traumas other than head trauma, malignancies and sepsis.

The limitation of our study is its being retrospective, absence of the interim values of patients before hospitalization and not being able to look at the survivors’ values. We have not been able to investigate in detail the antiplatelet agents, nonsteroidal anti-inflammatory drugs and smoking status known to affect MPV. Despite these limitations, the main strength of this study is the inclusion of a relatively large number of patients from a single center, in the same patient groups. ICU mortality rates are high. In studies, ICU mortality rates in Turkey are reported to be between 27% and 60%. In a meta-analysis study by Roquilly et al., mortality rates were found to be between 22.6% and 22.7% [7].

In our study, we found the rate of mortality was 19%. There are many scoring systems and laboratory studies that determine the prognosis of ICU patients. We examined the changes in the final values ​​of PLT, MPV and RDW in ICU admission and deaths from simple, economical and accessible examinations, which were looked at daily, according to the diagnosis in all intensive care patients and ICU admissions. There was a statistically significant decrease in PLT, increase in MPV and RDW in all groups. We believe that decrease in PLT, increase in MPV and RDW is a prognostic factor for mortality.

Thrombotic events and inflammation may also alter the extent of thrombocytosis, which can be detected in routine blood cell analysis by evaluating the MPV. MPV is also affected by platelet aging and depends on the balance between production and destruction. The degree of inflammation and changes in MPV appear to be interrelated in several clinical conditions. However, the effect of this is controversial. Since there is a strong inverse correlation between platelet count and MPV in healthy individuals, trends in platelet count should be considered when evaluating MPV [8-10].

There are many studies investigating the relationship between mortality and MPV in the intensive care unit. Altun et al. reported that MPV did not correlate with mortality in their study [11]. Tajarernmuang et al. in their meta-analysis reported that MPV of patients admitted to ICU was not associated with mortality, but in some studies gradual increase of MPV was associated with mortality [12]. In another study, the mean MPV level discharge was higher than the initial MPV level in patients who died, whereas in the living group MPV levels were decreased [13]. It was compatible with our work.

RDW, which reflects erythrocyte morphology, is frequently used in the differential diagnosis of anemia. However, systemic inflammation, nutritional disorders, ineffective erythropoiesis and bone marrow dysfunction may also lead to an increase in RDW [6,14]. It has been suggested that RDW increase is associated with impaired erythropoiesis due to underlying metabolic abnormalities such as oxidative stress, inflammation, malnutrition, dyslipidemia, erythrocyte fragmentation and impaired erythropoietin function [6,15,16]. High RDW levels have been reported to play an important role for short- and long-term prognosis [6]. Although the underlying mechanism is not fully understood, it has been shown that there is an independent relationship between increased mortality and RDW levels in critically ill patients [17]. RDW is defined as a prognostic marker in cardiovascular diseases [18]. It was also reported that the use of RDW level and RDW change in patients with heart failure might be useful in predicting prognosis [19]. The current study also supports this.

A study by Özdoğan et al. found that RDW increase was significantly associated with mortality in community-acquired intra-abdominal sepsis patients [20]. RDW showed mortality predictions as a strong predictor as APACHE II score. Many pathological processes including inflammation, oxidative stress, malnutrition, cardiovascular diseases, heart failure and increased RDW in renal failure may be a prognostic marker. RDW value can be a prognostic factor in inflammation, many pathological status, oxidative stress, malnutrition, cardiovascular disease, cardiac failure and kidney failure.

In the current study, the number of cardiac patients was significantly high in respiratory failure, cardiac causes, and traumas other than head trauma, malignancies and sepsis.

## Conclusions

In the study, the initial PLT, MPV and RDW admission values and before death PLT, MPV and RDW values were compared in all patients hospitalized in the ICU. In addition, the study, which was examined individually according to the diagnosis in the ICU, showed that the decrease in PLT and the increase in MPV and RDW were statistically significant. We think that the decrease in PLT and the increase in MPV and RDW are a prognostic factor for mortality. We think that with a prospective study, the cut-off value of the MPV and RDW can be determined with a decrease in PLT value.
